# Vegetation type influences particulate organic matter storage along a low Arctic vegetation gradient

**DOI:** 10.1007/s10533-025-01294-9

**Published:** 2025-12-08

**Authors:** Lewis Sauerland, Rica Wegner, Andrei Moise, Lukas Kohl, Jenie Gil, Birgit Wild

**Affiliations:** 1 Department of Environmental Science, Stockholm University, Stockholm, Sweden; 2 Bolin Centre for Climate Research, Stockholm University, Stockholm, Sweden; 3 Department of Environmental and Biological Sciences, University of Eastern Finland, P.O. Box 1627, 70211 Kuopio, Finland

**Keywords:** Permafrost soils, Terrestrial arctic ecosystems, Physical soil fractionation, Arctic shrubification, Carbon cycling, Lignin phenol biomarker

## Abstract

Permafrost soils constitute a large part of the terrestrial carbon pool that is vulnerable to future climate warming. Continued warming of the low Arctic is also leading to the encroachment of large shrubs and trees into tundra ecosystems with effects on microbial community composition, organic matter cycling and physical soil parameters. To date it is still largely unknown how such vegetation shifts affect soil organic matter cycling in permafrost soils on short and long timescales. Here, we investigated differences in soil organic matter properties under graminoid tussock (*Eriophorum vaginatum*), birch shrub (*Betula glandulosa*), spruce tree (*Picea mariana*) and alder shrub (*Alnus viridis*) vegetation by density fractionation and subsequent measurements of organic carbon, total nitrogen, δ^13^C, and lignin phenol biomarker contents. Particulate organic matter constituted 1.3–11.3% of soil weight and stored between 29 and 89% of the total soil lignin, 12–60% of organic carbon and 6–40% of total nitrogen. The contribution of particulate organic matter generally decreased with soil depth. Soils under *Alnus viridis* showed significantly higher amounts of particulate organic matter and stored more lignin, organic carbon and total nitrogen in particulate form in all soil depths. Sites dominated by *Eriophorum vaginatum* exhibited higher lignin content and lower degradation state in the subsoil, which was associated with water saturation and low active layer depth. We conclude that the effect of vegetation changes on soil organic matter cycling is dependent on plant species with the encroachment of *Alnus viridis* shrubs potentially increasing the deposition of particulate organic matter into permafrost soils.

**Supplementary Information:**

The online version contains supplementary material available at 10.1007/s10533-025-01294-9.

## Introduction

Rapid Arctic warming is affecting permafrost soils which store ca. 500 Pg of carbon within the top 1 m (Hugelius et al. 2014). Such warming will weaken the mechanisms that have thus far protected soil organic matter (SOM) in permafrost soils from degradation, including freezing, low temperatures and water saturation. This is proposed to lead to an increased remineralisation of SOM to carbon dioxide and methane, further exacerbating climate change (Schuur et al. 2015). Another effect of climate warming is the northward expansion of the treeline and tall deciduous shrubs, which has been observed for many regions of the Arctic (Harsch et al. 2009; Heijmans et al. 2022; Mekonnen et al. 2021). For example, on-site observations in northwestern Canada have shown a 55% increase in tall shrub coverage on the Tuktoyaktuk coastal plain within 33 years (Moffat et al. 2016). Trees and large shrubs will likely expand even further north in the future, as was also the case during the warmer mid-Holocene (Bigelow et al. 2003). Such vegetation changes can accelerate SOM degradation by increasing soil temperature through snow trapping and decreased albedo (Sturm et al. 2005) or via increased rhizodeposition leading to a stronger rhizosphere priming effect (Street et al. 2020). On the other hand, higher above- and below ground litter production might increase SOM content by the input of fresh organic matter into the soil (Blume-Werry et al. 2019; Cornelissen et al. 2007; Feng et al. 2013; Wang et al. 2016). So far it remains unclear whether changes to plant cover and site characteristics favour organic matter storage or losses.

One approach for investigating the fate of plant residues in the soil is the analysis of lignin which is a main component of plant biomass (Kögel-Knabner 2017). Total lignin content is utilized to quantify the contribution of plant biomass to soil organic matter and ratios of individual lignin phenols are used to assess the degradation state of lignin (Jex et al. 2014). Previous studies investigating lignin phenol biomarkers in Siberian permafrost soils across latitudinal and continentality gradients found lower lignin phenol concentrations and higher lignin degradation state in soils with deeper active layers, higher temperatures and moderate soil moisture (Dao et al. 2022, 2023). Active layer depth and temperature of permafrost soils are likely to increase with continued climate warming, thus improving the conditions for SOM and lignin decomposition.

Another commonly used approach for investigating the fate of plant residues in soil is by separating bulk soil into fractions of differing density (Lützow et al. 2007). Thereby, bulk soil is separated into particulate residues outside of aggregates (free particulate organic matter; ‘free fraction’), particulate residues encapsulated within soil aggregates (occluded particulate organic matter; ‘occluded fraction’) and amorphous organic matter bound to mineral surfaces (mineral associated organic matter; ‘mineral fraction’) (Lützow et al. 2008). The potential degradability is considered to decrease from free to occluded to mineral fraction, based on the assumption that occlusion and mineral-association are the main processes inhibiting SOM degradation (Lützow et al. 2007, 2008). Accordingly, SOM in the free and occluded fractions are considered a part of the active and intermediate SOM pool, whereas SOM in the mineral fraction is considered part of the passive SOM pool (Lützow et al. 2008). Since permafrost soils might contain a higher percentage of SOM in the particulate fractions compared to temperate soils (Diochon et al. 2013), the susceptibility of permafrost SOM to re-mineralisation is potentially also higher if other stabilizing mechanisms such as low temperatures and anoxia decrease. To date, further research is still needed to understand how differing vegetation types affect the amount of SOM stored in different fractions of permafrost soils and whether these relations change with observed shifts in vegetation.

In this study we investigated differences in organic matter stabilisation in permafrost soils in the low Arctic Tundra of northwestern Canada. Soil samples were taken from four mineral soil sites under different dominant vegetation cover typical for regional vegetation patterns including graminoid tussock, low shrub, large shrub and coniferous trees and were density fractionated into free, occluded and mineral fractions. All fractions were analyzed for elemental and isotopic composition and lignin phenols. The percentages of organic carbon, nitrogen and lignin stored within each fraction were compared between soil depths and sites. Additionally, the degradation state of SOM bound in each fraction was investigated using isotopic (δ^13^C), elemental (C/N) and molecular (lignin phenols) approaches. We hypothesized that 1) inter-site differences of organic matter properties are strongest in the litter and decrease with soil depth for bulk soil samples; 2) increased litter inputs at sites dominated by large shrubs and coniferous trees lead to higher contents of particulate organic matter, especially in the topsoil; 3) lignin is predominantly stored in particulate fractions, whereas larger proportions of carbon and nitrogen are stored in the mineral fraction.

## Materials and methods

### Study area and site selection

**Fig. 1:**
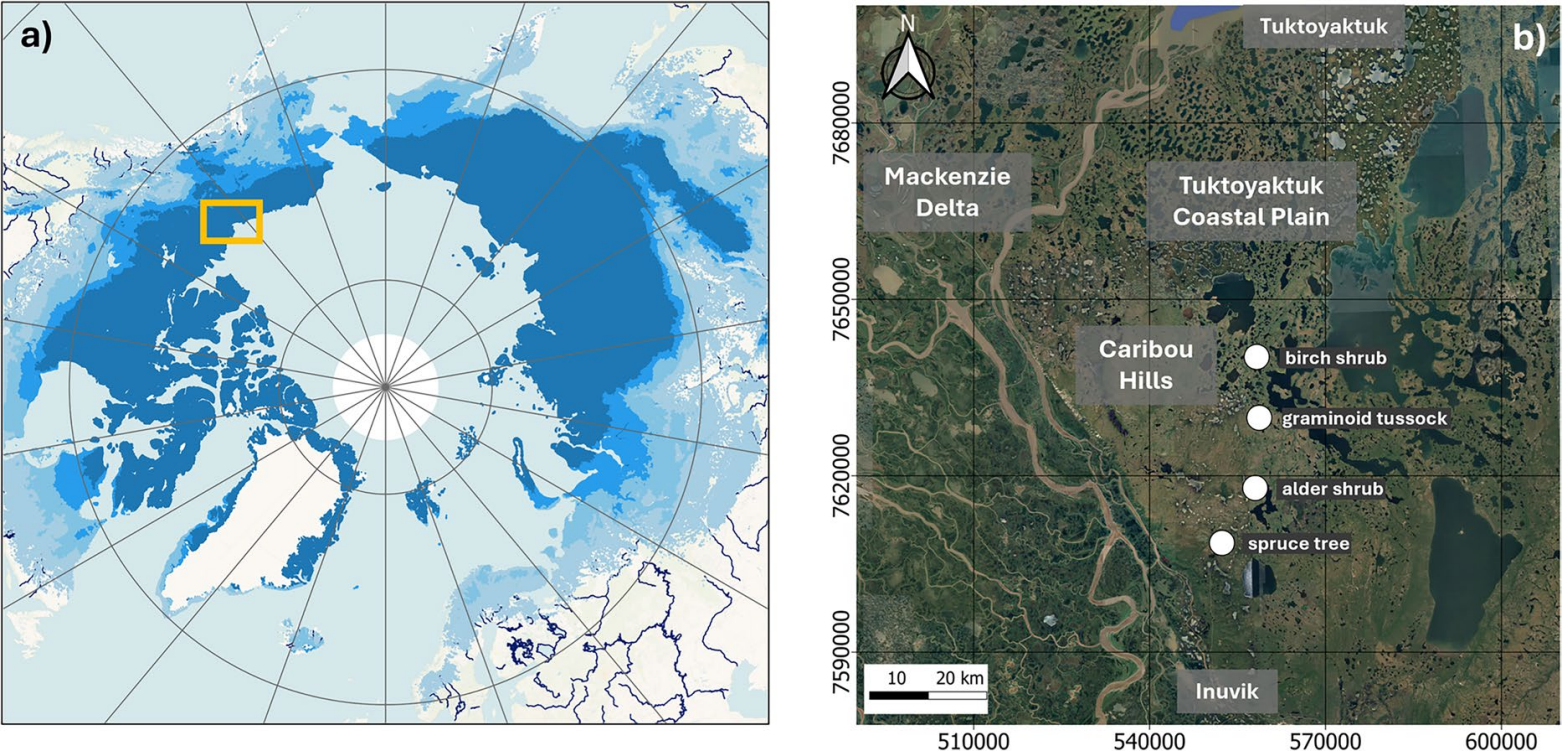
Location of the study area and sampling sites. **A** Shows the broader location of the study area within the Arctic, as indicated by the orange rectangle. The blue shading of the land indicates the permafrost distribution from continuous (dark blue) to sporadic (light blue). **B** Shows the locations of the individual study sites, which are labelled as: birch shrub, graminoid tussock, alder shrub and spruce tree. The approximate locations of the eco regions Tuktoyaktuk Coastal Plain, Caribou Hills and Mackenzie Delta are indicated with text boxes. Both maps were created using QGIS; the permafrost map for panel a was sourced from Obu et al. (2019) and the satellite imagery of panel b was sourced from Google Earth

The study area is located north of Inuvik in northwestern Canada (latitude: ca. 68.7° N; longitude: ca. 133.6° E) within the continuous permafrost zone of the low Arctic and is part of the ecoregions Caribou Hills and southern Tuktoyaktuk Coastal Plain (Fig. 1; Table S1). The area is characterized by rolling hills covered by fine grained glacial deposits intersected by drainage channels consisting of fluvial, limnic or organic deposits. A strong vegetation gradient is present with patchy forest tundra vegetation in the southern parts of the research area changing to stands of tall shrubs and finally to low shrub and tussock tundra ecosystems northwards (Ecosystem Classification Group, 2012). Field sampling was conducted during late August–early September 2022, close to the end of the vegetation period. Four sites were selected based on dominant vegetation cover while minimizing variability in other environmental properties (Fig. 1b). All sites were situated within 32 km and 0.28° latitude of each other, at 46–106 m altitude, on the top or flanks of ridges that were covered by hummocky mineral permafrost soils on fine-grained glacial deposits. All sites were well-drained, with Stagnic Turbic Cryosols based on WRB (Schad 2016).

Four different dominant vegetation types were selected to be representative of the vegetation found in the area: *Picea mariana* trees, tall *Alnus viridis* (syn. *Alnus alnobetula*) shrubs (1–4 m), *Betula glandulosa* shrubs (0.3–0.8 m), and tussocks dominated by *Eriophorum vaginatum* (Fig. S1). The four species differ in a range of properties that could influence SOM formation and stabilization. First, *P. mariana* is an evergreen tree, *A. viridis* and *B. glandulosa* are deciduous shrubs, and *E. vaginatum* is evergreen, perennial, non-woody, and tussock forming; this influences quantity and quality of leaf litter inputs. Second, the four plant species differ in root morphology and depth. *P. mariana*, *A. viridis* and *B. glandulosa* roots form perennial, woody roots that were largely within the top 20–30 cm of the soil at the field site. *E. vaginatum* grows thin, low-density roots to the permafrost table every year. Third, *P. mariana*, *A. viridis* and *B. glandulosa* are associated with ectomycorrhiza, and *A. viridis* additionally with nitrogen-fixing bacteria. *E. vaginatum* can be associated with dark septate endophytes and sporadically arbuscular mycorrhiza, as observed also at the field site (Wegner et al. 2025). These differences suggest that *P. mariana* and *B. glandulosa* take up nitrogen mainly from the shallow soil, *E. vaginatum* from the deeper soil, and that *A. viridis* relies on its nitrogen-fixing symbionts. This is supported by ^15^N values of leaves and roots of these plants at our field sites (Wegner et al. 2025). Finally, root exudates of *A. viridis*, *B. glandulosa* and *E. vaginatum* at our field sites were similar in release rates of total organic carbon and primary metabolites per fine root surface area, but differed in secondary metabolite composition (Wegner et al. 2025). The sites are further referred to as spruce tree, alder shrub, birch shrub and graminoid tussock site. The understory was composed of varying amounts of: *Vaccinium vitis-idaea*, *Empetrum nigrum*, *Rhododendron tomentosum*, *Rubus chamaemorus* and several other graminoid species.

### Sampling and sample preparations

For every site four individual hummocks were sampled as spatial replicates. At each replicate site a soil pit was dug until the bottom of the active layer (thaw depth 33–80 cm; Table S1), soil horizons identified, and representative bulk soil samples taken from each horizon. Soil temperature was measured in situ at different depths using a digital thermometer rod. Diagnostic soil horizons were identified and soils classified as Stagnic Turbic Cryosols based on the international soil classification system WRB (Schad 2016; Fig. S2). Three mineral soil horizons were identified at most profiles: organic matter enriched topsoil (Ah), well-aggregated and drained upper subsoil (Bw@) and cohesive, wet to water saturated lower subsoil (Bg@). Additionally, all profiles had a litter horizon (L) and a surface dry organic horizon of low to intermediate decomposition state (O). Bulk soil samples were homogenized and living roots removed on the day of sampling. Immediately afterwards the soil samples were frozen and kept continuously frozen until further processing. Leaf litter samples were taken from the dominant vegetation directly above each soil profile, dried for 72 h at 60 °C and stored dry and dark until further processing. Prior to laboratory analyses, bulk soil samples were dried at 105 °C for 72 h. For elemental and lignin analyses of bulk mineral soil, organic horizons and litter samples, subsamples were homogenized with a ball mill (MM400, Retsch GmbH, Germany). The rest of dried bulk mineral soil was sieved through 2 mm mesh sieve as preparation for density fractionation.

### Soil organic matter fractionation

The method used for separating SOM fractions via density fractionation is based on (Liebmann et al. 2023) with minor modifications. Briefly, the dried and sieved bulk soil was suspended in a sodium polytungstate solution with a density of 1.6 g cm^−3^ and the material suspended on top of the fluid was siphoned off. This material corresponds to free particulate organic matter. Afterwards, the left-over soil was disaggregated using an ultrasonicator (VCX 500, Sonics & Materials inc, USA) at 75% amplitude and a total energy input of 60 J mL^−1^. The material suspended on top of the sodium polytungstate solution after sonication was siphoned off and corresponds to occluded particulate organic matter. The left-over material with a density > 1.6 g cm^−3^ corresponds to the soil fraction containing mineral associated organic matter. For simplicity, the density fractions are further referred to as free fraction, occluded fraction, particulate fraction (POM; free + occluded fraction) and mineral fraction. Following separation, all three fractions were rinsed with deionized water until an electric conductivity of < 10 µS cm^−1^ was reached, frozen and freeze-dried. The freeze-dried samples were milled with a ball mill (see above) prior to elemental analyses and lignin phenol extraction. The recovery was calculated by dividing the sum of all three fractions (dry weight) by the initial sample dry weight and ranged between 80 and 94%. The percentage of each density fraction was calculated relative to the sum of all three density fractions as dry weight (e.g. free fraction/∑(free fraction + occluded fraction + mineral fraction) × 100). Additionally, the percentage of lignin, OC and TN stored in each respective fraction was calculated by normalizing the content of lignin, OC and TN measured for each fraction to the percentage of each fraction.

### Grain size, elemental and isotopic analysis

Sample preparation for grain size analysis consisted of combustion of organic matter at 400 °C for 12 h and subsequent ash removal by washing the combusted samples twice with deionized water. Grain size analysis was conducted using a laser diffraction analyzer coupled to a sample dispersion unit (Mastersizer 3000 and Hydro LV, Malvern Instruments Ltd). Samples were dispersed using sonication and by adding sodium metaphosphate to the dispersion unit. Grain size analysis was repeated five times per sample and results are given as mean values. The grain size data was classified according to the international soil classification system WRB as clay (< 2 μm), silt (2–63 μm), and sand (> 63 μm; Schad 2016).

Contents of organic carbon (OC) and total nitrogen (TN) were measured for all soil samples and δ^13^C was measured for all density fractionated samples. Therefore, dried and ground plant and soil samples were weighed into tin capsules. Acidic soil pH (< 6.0; Table S2) and lack of inorganic carbon, determined by selectively measuring acidified vs. non-acidified soil samples, indicated that the presence of carbonates could be excluded and thus samples were not acidified prior to analyses. Two different elemental analysers coupled to isotope ratio mass spectrometers were used. Bulk soil, litter and leaf samples were analysed at Stockholm University, Department of Environmental Science using an EA IsoLink–Delta V Plus system (Thermo Fisher Scientific, USA). Density fractionated soil samples were measured at the University of Eastern Finland, Kuopio using a Flash EA 1112–Delta XP Plus system (Thermo Finnigan, Germany). Organic carbon and total nitrogen contents are given in weight percentages relative to soil dry weight. For measurements at the University of Eastern Finland two reference materials were used for quality control. Wheat flour with certified δ^13^C (IVA Analysetechnik; Meerbusch, Germany) was used for isotope calibration and L-glutamic acid (USGS 40) was used as a check standard. For measurements at Stockholm University two certified reference materials were used. Low Organic Soil Standard (Certificate Nr.:324,704; δ^13^C VPDB = − 22.88 ± 0.40 ‰, OC w/w = 1.86 ± 0.14%, N w/w = 0.122 ± 0.018%; Elemental Microanalysis, UK) was used for isotopic calibration of all samples and for carbon content calibration of mineral soil samples and Peat Soil Standard (Certificate Nr.: 133,519; OC w/w = 15.95 ± 0.03%, N w/w = 1.29 ± 0.02%; Thermo Fisher Scientific, USA) was used for calibration of organic horizon and litter samples. The δ^13^C values in this study are given in ‰ relative to Vienna Pee Dee Belemnite. Method precision, determined as the relative standard deviation from repeated measurements of quality control standards throughout an analysis sequence, was 2.49%, 14.71% and 0.27 ‰ (%C, %N and δ^13^C) for the Kuopio system and 1.9%, 2.4% and 0.2 ‰ (%C, %N and δ^13^C) for the Stockholm system.

### Lignin phenol extraction and analysis

The method used for the extraction, purification and analysis of lignin phenols is based on Goñi and Montgomery (2000) with minor modifications described in Martens et al. (2019). In summary, lignin phenols were liberated from the soil via alkaline copper oxide oxidation using a microwave digestion system. After acidification, the lignin phenols were extracted with ethyl acetate and the extracts evaporated and re-dissolved in pyridine. Two internal standards, ethylvanillin and trans-cinnamic acid, were used to determine the recovery which ranged between 34–87 and 75–100%, respectively. Separation, identification and quantification of individual lignin phenol monomers was accomplished using a gas chromatography mass spectrometry system (7820A, Agilent Technologies, USA).

Total lignin phenol content was used as a proxy for the contribution of structural plant litter to SOM. It was calculated as the sum of syringyl, cinnamyl and vanillyl phenols in mg and normalized to one-gram dry soil (mg g^−1^ DW) and one-gram organic carbon (mg g^−1^ OC). To investigate the decomposition state of lignin a commonly used degradation proxy was calculated as the ratio of vanillic acid over vanillin (Vd/Vl) (Jex et al. 2014). Proxy values increase with progressing lignin degradation state. Average method uncertainty, determined as the relative standard deviation after extracting and analysing replicates of the same soil sample (n = 3), was 14.7% for lignin content and 7.3% for the degradation proxy.

### Statistics

Statistical analyses were performed in R (R Core Team 2024). Linear mixed effects models (lmer function in the lme4 package; Bates et al. 2015) were used to test for differences in measured parameters, with sites and soil horizons as fixed effects for bulk soil parameters, as well as sites, soil horizons and soil fractions for fractionated soil samples. Soil plot was included as random factor. Interactive effects were included, but removed from the model in cases where they were not significant at p < 0.05. All data were log transformed before model fitting as this improved data distribution. Normal distribution of residuals was confirmed using Q-Q-plots, and homoscedasticity by plotting residual vs fitted values. The emmeans function in the emmeans package (Lenth 2025) was used as a post hoc test. The significance threshold for comparisons is defined as p = 0.05, with p < 0.05 indicative of significant differences between mean values. Values slightly above the significance threshold (0.05 < p < 0.1) are referred to as marginally significant. All significance values used for this publication are given in the Supplementary Information 2 (SI2).

## Results

### Abiotic soil properties

Analysis of a range of abiotic soil properties confirms the comparability of the four sites regarding texture and soil temperature regime (Tables S1–S2). All soils were classified as silt loam according to the WRB (Schad 2016). Clay, silt and sand content showed significant but small differences among sites. The alder shrub and spruce tree sites (mean ± standard deviation: 19 ± 3%) had significantly higher clay content than the birch shrub and graminoid tussock sites (13 ± 2%; SI2). Silt content was higher at the graminoid tussock (78 ± 1%) than at the other sites (73 ± 3%), and sand content was higher at the birch shrub (14 ± 4%) than at the alder shrub and spruce tree sites (8 ± 3%). Active layer thickness was not significantly different across the four sites. Soil temperature was marginally significantly (p = 0.083) lower under graminoid tussock than under spruce trees, by on average 1.4 °C.

### Litter and bulk soil organic matter properties

**Fig. 2:**
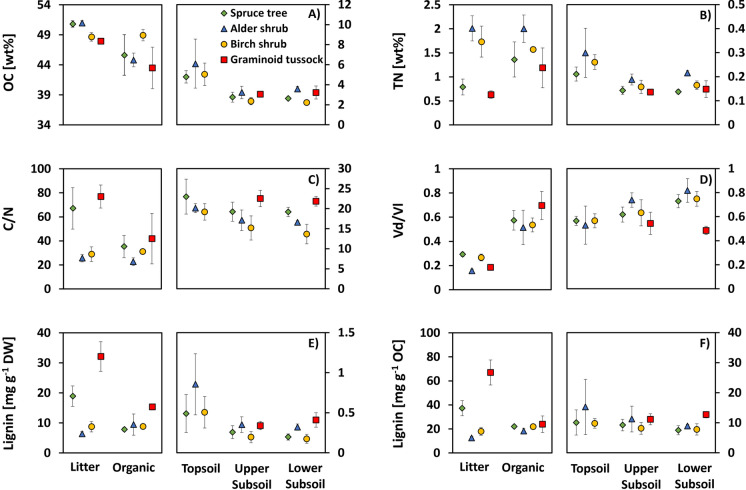
Bulk soil organic matter properties. Organic carbon content (OC) and total nitrogen content (TN) in weight percent relative to soil dry weight (**A** and **B**), organic carbon to total nitrogen ratio (C/N; **C**), the ratio of vanillic acid over vanillin (Vd/Vl; **D**) and the lignin content normalized to one gram dry weight and one gram organic carbon (Lignin; **E** and **F**) are shown for five soil horizons including litter, dry organic horizon (organic), topsoil, upper subsoil and lower subsoil. Values for litter and organic horizons are plotted in separate windows to the mineral soil horizons to enable a separate scaling of the y axis. Values are shown for four different main sites that were defined based on their dominant vegetation cover: spruce tree (green diamonds), alder shrub (blue triangles), birch shrub (orange circles) and graminoid tussock (red squares)

Leaf litter showed significant differences in stoichiometry and chemical composition among plant species. *A. viridis* and *B. glandulosa* litter had significantly higher TN, lower C/N and lower lignin content compared to *P. mariana* and *E. vaginatum* (Fig. 2; Tables S3, SI2). These differences were weaker in the organic horizon, as TN, lignin and C/N of *P. mariana* and *E. vaginatum* approached those of *A. viridis* and *B. glandulosa*.

From organic to mineral soil horizons, OC, TN and lignin content showed consistent decreases with soil depth (Fig. 2a, b, e, f; Table S2). Linear mixed effects models showed significant (p < 0.05) decreases between litter and organic horizons compared to mineral horizons as well as between topsoil and subsoil horizons, but not between subsoil horizons (SI2). The lignin degradation state (Vd/Vl; Fig. 2d; Table S2) increased with soil depth at any given site except graminoid tussock. This increase in Vd/Vl was significant between litter and all other horizons at all sites, and between organic horizon and topsoil compared to subsoil horizons in many cases (SI2). A significant decrease of C/N with soil depth was observed between organic (L and O) and mineral soil (SI2), except for alder shrub where differences were only significant between litter and subsoil horizons. At the graminoid tussock site lignin content increased and degradation state decreased between upper and lower subsoil horizons, but these differences were not statistically significant (p > 0.1).

Inter-site comparisons showed significant differences between sites for bulk soil parameters (Table 1). Alder shrub showed significantly higher TN compared to graminoid tussock and spruce tree in most soil horizons (p < 0.1 except in the upper subsoil compared to spruce tree). The graminoid tussock site exhibited higher lignin content and C/N and lower lignin degradation state (Vd/Vl) than all other sites. Specifically, lower subsoil horizons showed significant differences in Vd/Vl between graminoid tussock and all other sites, and in lignin content compared to birch shrub and spruce tree sites (SI2).

**Table 1:**
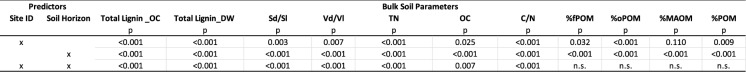
Linear mixed effects model output for bulk soil parameters

Significance values are given for the analysis of variance using Site ID and Soil Horizon as predictors, and their interactive effects

### Particulate and mineral-associated soil fractions

**Fig. 3:**
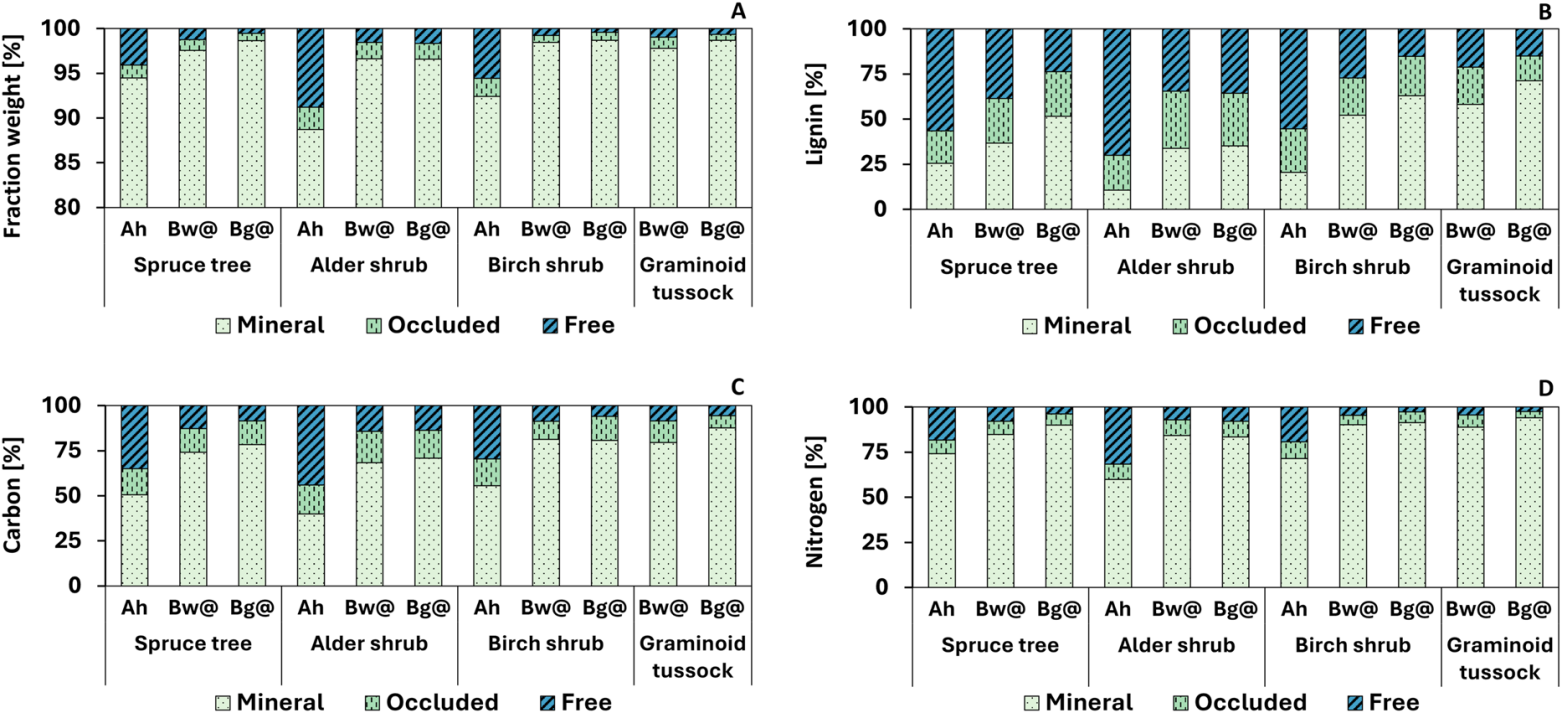
Storage of organic matter in density fractions as percentage of total. **A**–**D** Show fraction weight, total lignin (lignin), organic carbon (carbon) and total nitrogen (nitrogen) for three density fractions: mineral fraction (Mineral), occluded fraction (occluded) and free fraction (free). Sites are named according to the dominant vegetation type as spruce tree, alder shrub, birch shrub and graminoid tussock site. Three soil horizons are abbreviated as Ah (topsoil), Bw@ (upper subsoil) and Bg@ (lower subsoil)

Particulate organic matter made up between 1.3 and 11.3% of total soil weight for all samples (Table S2). Content of POM showed high variability between sites and with soil depth (Fig. 3a). Topsoil horizons had significantly higher POM content than both subsoil horizons whereas no difference was observed between upper and lower subsoil.

The percentage of lignin, OC and TN bound in POM showed a decreasing trend with soil depth for all sites (Fig. 3b–d; Tables S4–S6). Linear mixed effects models showed significant differences in lignin, OC and TN storage between soil horizons for the free fraction, in lignin for the mineral fraction and largely no differences for the occluded fraction (Table 2; SI2). Overall, between 29 and 89% lignin, 12 and 60% organic carbon and 6 and 40% nitrogen was stored in POM. Associated depth trends showed that in the topsoil approximately 75% of lignin, 50% of organic carbon and 30% of nitrogen was stored in POM, which decreased to ca. 40% lignin, 20% organic carbon and 10% nitrogen in the lower subsoil (Tables S4–S6).

**Table 2:**
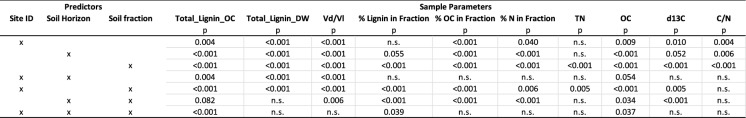
Linear mixed effects model output for soil fraction properties

Significance values are given for the analysis of variance using Site ID, Soil Horizon and Soil Fraction as predictors, and their interactive effects

The alder shrub site contained significantly more POM across all horizons compared to other sites (Fig. 3a; SI2). This was also observed for the lower subsoil in which the amount of POM was ca. 2.5 times higher at the alder shrub than at all other sites (3.4 wt% versus ca. 1.3 wt%). Accordingly, the alder shrub site also had the highest percentage of lignin, organic carbon and nitrogen stored in POM in all soil depths.

**Fig. 4:**
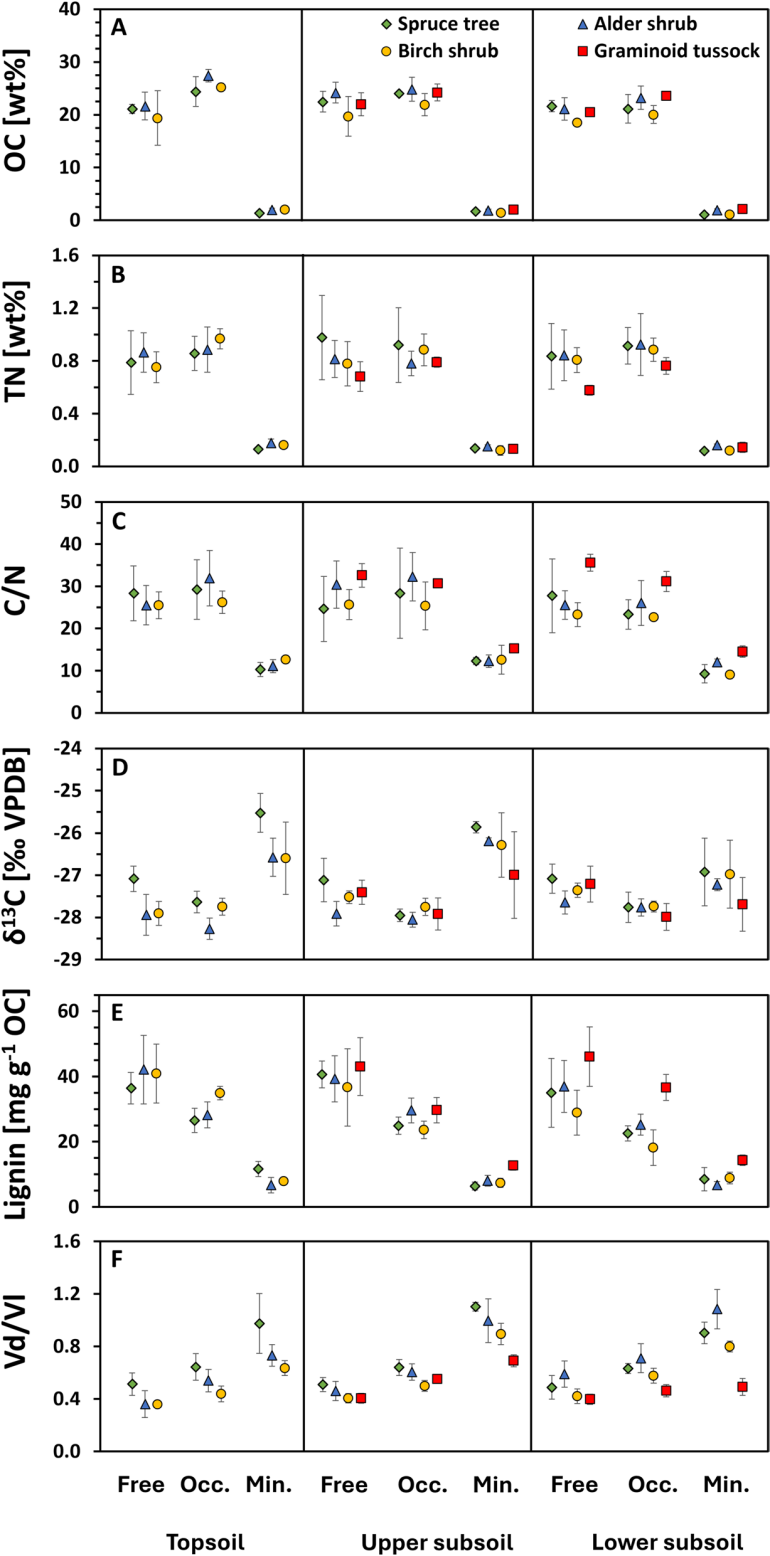
Soil organic matter properties of different density fractions. Data points represent mean values and error bars indicate the associated standard deviation. Organic carbon content (OC) and total nitrogen content (TN) are given in weight percent and shown in **A** and **B**. **C** Depicts the organic carbon to total nitrogen ratio (C/N), which decreases with advancing organic matter degradation. **D** Shows the stable isotope composition of organic carbon given as δ^13^C in ‰ relative to VPDB; increasing values indicate progressing organic matter degradation state. Lignin content (Lignin) normalized to gram organic carbon is given in mg g^−1^ OC (**E**). **F** Depicts the lignin degradation state proxy vanillic acid over vanillin (Vd/Vl); increasing values correspond to a higher lignin degradation state. Values are shown for four different main sites that were defined based on their dominant vegetation cover: spruce tree (green diamonds), alder shrub (blue triangles), birch shrub (orange circles) and graminoid tussock (red squares)

Particulate and mineral fractions differed in organic matter composition and degradation state (Fig. 4). Contents of lignin, OC and TN were significantly lower in the mineral fraction compared to the free and occluded fraction in all sites and horizons (SI2; Fig. 4a, b, e; Tables S4–S6). Cross-site comparisons revealed that free, occluded and mineral fractions showed some significant differences in lignin, OC and TN (Table 2; SI2). Most notably, the graminoid tussock site exhibited significantly higher lignin content compared to all other sites in the mineral fraction, and for the lower subsoil also in the occluded fraction.

The degradation state of SOM assessed using Vd/Vl, C/N and δ^13^C, showed significant differences between fractions. Specifically, free and occluded fractions had consistently significantly lower Vd/Vl and higher C/N compared to mineral fractions (SI2). An exception to this was found for the lower subsoil of the graminoid tussock site, which did not exhibit significant differences between fractions. The δ^13^C values were significantly lower in the free and occluded compared to the mineral fractions for topsoil and upper subsoil horizons at all sites (except graminoid tussock, upper subsoil, free vs mineral fractions). The occluded fraction showed lower lignin concentrations (normalized by OC) and higher Vd/Vl ratios than the free fraction (Fig. 4).

## Discussion

This study aimed to investigate how SOM is stored and stabilized in mineral permafrost soil and whether shifts in vegetation are likely to affect this. To address this overarching question, we first assessed if litter inputs from encroaching plant species have different OM properties and whether variability in litter properties, together with differences in belowground properties such as root morphology, rooting depth, and root-microbial symbioses, lead to contrasting contents of organic carbon, nitrogen and lignin in the underlying bulk soil. Dissecting SOM quantity and quality in particulate and mineral-associated soil fractions then allowed us to elucidate the possible impact of changing plant cover on the SOM storage in more or less labile fractions.

### Litter and bulk soil properties differ among vegetation types

Leaf litter from *B. glandulosa* and *A. viridis* exhibited significantly higher TN content, lower C/N values and lower lignin contents compared to *P. mariana* and *E. vaginatum* litter (Fig. 2). The differences especially between *A. viridis* and other plant species were expected, since it is known that *A. viridis* litter contains larger quantities of TN due to its symbiosis with nitrogen fixing bacteria (Bühlmann et al. 2016). Total nitrogen content and C/N of *A. viridis*, *P. mariana* and *E. vaginatum* litter were also comparable to previously reported values for the same species or genera (Hobbie 1996; Lorenz et al. 2000; Ramm et al. 2022). *B. glandulosa* litter exhibited higher TN and lower C/N than low birch shrubs (Hobbie 1996), but comparable values to larger birch shrubs (Buckeridge et al. 2010).

Contents of OC, TN and lignin generally decreased from organic layer to topsoil and subsoil, and the compositional differences observed for plant litter largely diminished with soil depth (Fig. 2). Nevertheless, the alder shrub site showed significantly higher TN values (except against birch shrub), and the graminoid tussock site higher contents and a lower decomposition state of lignin in the lower subsoil compared to other sites (Fig. 2, SI2). The decrease in C/N, OC, TN and lignin contents from litter to the subsoil is consistent with previous investigations in Arctic tundra ecosystems (Dao et al. 2018; Fouché et al. 2014). This is likely due to a combination of decreasing leaf and root litter inputs with soil depth, influence of understory vegetation litter and a relative enrichment of nitrogen and depletion of organic carbon and lignin with progressing aerobic decomposition of SOM. Higher soil C/N, lignin contents and lower lignin degradation state in the subsoil at the graminoid tussock site are comparable to previous studies in Siberia at sites with similar vegetation cover and physical site characteristics (Dao et al. 2022). Possible explanations for these trends, besides litter composition, are fresh litter input from deep annual roots to the subsoil, low subsoil temperature (Table S1) and observed subsoil water saturation at the graminoid tussock site of this study, which are likely to inhibit organic matter and especially lignin decomposition (Dao et al. 2023). The consistent patterns of lignin accumulation in graminoid tussock subsoils in this and previous studies could thus be a direct effect of graminoid tussock vegetation, or of the abiotic conditions favouring its establishment.

The analysis of litter and bulk soil properties showed that even though plant species specific differences in leaf litter composition diminished with soil depth, alder shrub and graminoid tussock site exhibited consistently different SOM properties. In order to further assess the potential effects of vegetation types on SOM storage and stabilisation, it is necessary to consider the percentage of SOM stored in physical soil fractions and their respective properties.

### Soils under large alder shrubs store more organic matter in particulate fractions

The physical separation of bulk soil into the free, occluded and mineral fractions showed that particulate organic matter (free + occluded fraction) makes up between 1.3–11.3 wt% of total soil weight and that large percentages of lignin, organic carbon and nitrogen are stored in the POM fraction (Fig. 3). As hypothesized, POM content and percentages of lignin, organic carbon and nitrogen stored in POM were higher in the topsoil than subsoil (Fig. 3). This likely reflects the decreasing influence of above-ground litter with depth, as well as decreasing inputs of root litter with lower rooting intensity in the deep soil (Blume-Werry et al. 2023).

Confirming our hypothesis, the percentages of lignin, OC and TN in each fraction showed that lignin is primarily stored in POM, whereas nitrogen is primarily stored in the mineral fraction (Fig. 3b, d). Organic carbon exhibited an approximately equal distribution between POM and mineral fraction in the topsoil and a dominance of the mineral fraction in the subsoil (Fig. 3c). Since lignin is exclusively produced by vascular plants and plant litter exhibits higher C/N than microbial biomass, lignin and OC storage are strongly coupled to the particulate fraction. Nitrogen, on the other hand, is to a larger extent derived from microbially re-worked SOM with lower C/N and stored in the mineral fraction (Bingham & Cotrufo 2016). This is further supported by previous findings of Dao et al. (2018) that showed higher microbial-derived neutral sugars in the mineral fraction compared to the particulate fraction. Overall trends observed for lignin, OC and TN storage in particulate versus mineral fractions were consistent with the general assumption that permafrost soils contain larger amounts of poorly degraded particulate plant residues compared to other terrestrial ecosystems (Ping et al. 2015).

In comparison with the other three dominant vegetation types, soils under *A. viridis* exhibited significantly higher POM content across all horizons, with ca. 2.5 times higher POM content in the lower subsoil (Fig. 3a). This is partially in line with our hypothesis that encroaching large *A. viridis* shrubs would lead to higher POM inputs but contradicts our assumption that these differences would be highest in the topsoil. Possible reasons for this observation are higher primary production and deeper rooting depth and intensity of *A. viridis* plants. It is likely that *A. viridis* plants have higher primary production than the dominant vegetation of other sites due to their larger size (1–4 m) and lower nutrient limitation because of nitrogen fixation and phosphorous supply from the young mineral soil substrate (Benecke 1970; Salmon et al. 2019). This is further supported by previous findings showing highest leaf area index values at shrub sites compared to heath, tussock and wetland sites and increased biomass growth for large not nitrogen fixing shrubs after nitrogen and phosphorous addition (DeMarco et al. 2014; Williams & Rastetter 1999). Plant roots were observed in subsoil horizons at the alder shrub site which, in combination with higher primary production, could increase root litter inputs into the subsoil. To what extent higher POM contents stem from above-ground or below-ground litter inputs cannot be determined, but it is likely that topsoil POM is more strongly influenced by above-ground litter, whereas subsoil POM is increasingly influenced by root litter. In line with this argumentation, the lack of subsoil POM at the spruce tree site might be coupled to a shallow rooting profile known for *P. mariana* and generally low primary production rates (Lamhamedi & Bernier 1994). To our knowledge there are no other publications that investigated physical soil organic matter fractions under *A. viridis*, which does not allow us to put the data into direct context.

The high OC and TN storage in POM (12–60% and 6–40%, respectively) and associated depth trends observed here are in line with previous publications of some Arctic ecosystems (Diochon et al. 2013; Prater et al. 2020). Opposing this, Gentsch et al. (2015a) and Gentsch et al. (2015b) estimated that only ca. 19% of OC was stored in the light fraction at their field sites across Siberia, which is approximately equivalent to the particulate fraction defined in this study. These discrepancies indicate a high variability in OC storage and importance of particulate and mineral fractions across Arctic soils. Possible reasons for these discrepancies could be that vegetation cover, soil type and soil texture were different between study sites.

In conclusion, our results showed that high percentages of soil organic matter are stored in particulate fractions with the alder shrub site having the highest POM storage especially in the subsoil. In order to assess the composition and degradation state of these SOM stocks it is necessary to evaluate the SOM properties of these different fractions.

### Organic matter properties of particulate fractions are distinct from the mineral fraction

The analysis of density fraction properties showed that lignin, OC and TN contents decreased between particulate and mineral fractions (Fig. 4). This is both in line with our expectations and with previous findings that assessed properties of density fractionated soils (Dao et al. 2022; Prater et al. 2020). A likely reason for this observation is that the majority of organic matter inputs into soils primarily stem from plant biomass (Kögel-Knabner 2017) and these contribute more strongly to labile particulate organic matter (Ping et al. 2015). By contrast, mineral-associated organic matter is to a larger extent derived from microbial biomass (Angst et al. 2021). Whereas differences of all analysed parameters between the free and mineral fractions of a given horizon were consistently significant, differences between free and occluded fractions were less pronounced (SI2). Lower lignin concentrations (normalized by OC) and higher Vd/Vl ratios of the occluded than the free fraction however suggest a more advanced decomposition state of lignin.

Inter-site comparisons showed significantly lower lignin degradation state in the subsoil of the graminoid tussock site compared to other sites. Density fractionation revealed that while the lignin degradation state of the free fraction was similar at the graminoid tussock compared to the other sites, lignin in the mineral fraction was substantially and significantly less degraded with Vd/Vl ratios only slightly above the free fraction (Fig. 4, SI2). This could indicate that water saturation and shallow active layer depth at the graminoid tussock site led to a higher conservation of organic matter and less differentiation between soil fractions. Water saturation and lower temperatures might thus supersede spatial inaccessibility and mineral association as constraining factors on organic matter stabilisation. This inference is in line with previous studies investigating climatic trends in organic matter storage and lignin degradation (Dao et al. 2022, 2023; Schuur et al. 2015).

### Implications for future climate and vegetation changes in the Arctic

The findings of this investigation indicate an influence of both biotic and abiotic parameters on SOM stabilization. Water-saturated subsoil horizons under *E. vaginatum* exhibited higher lignin content and lower lignin degradation state in bulk soil and physical fractions than other sites. This implies that future changes to soil hydrology and to vegetation cover would both affect SOM storage. Deepening of the active layer and improved drainage of mineral soils would subject poorly degraded subsoil POM to aerobic decomposition. Additionally, changing vegetation patterns on drained mineral soils would further affect SOM storage and stocks by modulating the input of particulate organic matter and thereby mainly affecting the fast carbon pool. It is uncertain what the net effect of these counter-acting processes would be on SOM storage in mineral permafrost soils.

Our findings also suggest that a progressing encroachment of large *A. viridis* shrubs into Arctic tundra ecosystems could affect soil organic matter storage to a larger extent than other encroaching species such as *B. glandulosa* and *P. mariana* by increasing the proportion of SOM stored in particulate fractions. Soils under large *A. viridis* shrubs contained higher amounts of particulate organic matter with the largest difference to other vegetation types in subsoil horizons. There are several potential implications for future changes to Arctic ecosystems from these observations. If particulate organic matter stocks are not just increased in organic and topsoil horizons, but also in subsoil horizons, this could mean that these increased subsoil inputs are more protected from remineralisation and disturbances such as wildfires, animals and erosion. This would have a counter-acting effect on the permafrost carbon feedback on short time scales. It is not possible to say whether this increased subsoil POM storage is likely to gradually increase or be re-mineralized with increasing temperatures and thaw depth. Increased inputs of labile root litter into subsoil horizons could also lead to priming effects resulting in increased remineralisation of more stabilized nitrogen rich organic matter bound in the mineral fraction. That elevated POM contents were only seen at sites dominated by *A. viridis* and not at sites dominated by *B. glandulosa* or *P. mariana* indicates that changes to SOM storage are likely specific to plant species. Thus, potential effects on SOM storage resulting from the encroachment of large shrubs and trees into Arctic tundra ecosystems will be strongly dependent on plant species type and changes to physical soil conditions.

## Supplementary Information

Below is the link to the electronic supplementary material.

Supplementary file1 (DOCX 6403 KB)

Supplementary file2 (XLSX 24 KB)

## Data Availability

The datasets generated for and used in this study are publicly accessible in the Bolin Centre Database (link will be added during production).
