# Supplementary material for: Vegetation type influences particulate organic matter storage along a low Arctic vegetation gradient
Source: Biogeochemistry. 2025 Dec 8;168(6):101. doi: 10.1007/s10533-025-01294-9 (PMC12705800; doi:10.1007/s10533-025-01294-9)
Supplement: Supplementary file 1 — Supplementary file1 (DOCX 6403 KB) [file 10533_2025_1294_MOESM1_ESM.docx]

Supplementary Information 1 of

**Vegetation type influences particulate organic matter storage along a low Arctic vegetation gradient**

Lewis Sauerland^1,2^, Rica Wegner^1,2^, Andrei Moise^1^, Lukas Kohl^3^, Jenie Gil^3^, Birgit Wild^1,2^

^1^ Department of Environmental Science, Stockholm University, Stockholm, Sweden

^2^ Bolin Centre for Climate Research, Stockholm University, Stockholm, Sweden

^3^ Department of Environmental and Biological Sciences, University of Eastern Finland, P.O. Box 1627, 70211 Kuopio, Finland

*Correspondence to*: Lewis Sauerland ([Lewis.Sauerland@aces.su.se](mailto:Lewis.Sauerland@aces.su.se)); Birgit Wild ([Birgit.Wild@aces.su.se](mailto:Birgit.Wild@aces.su.se))

# Contents

Figures S1 – S2

Tables S1 – S6

# Introduction

This document contains additional information for the above-mentioned manuscript including figures, photographs and data tables. The supplementary information is divided into 3 parts.

**Part S1** provides additional information on the investigated research sites including general site characteristics (Table S1), photographs of each research site (Figure S1) and a photograph of an exemplary soil profile (Figure S2).

**Part S2** lists individual values for all soil properties analysed for the main manuscript and is divided into bulk mineral soil (Table S2), litter and organic horizons (Table S3), free fraction (Table S4), occluded fraction (Table S5) and mineral fraction (Table S6).

**Part S3** gives individual significance values to all statistical tests used within the manuscript. This data is submitted as a separate excel-file named ‘Supplementary Information 2’.

**Table S1:** Site properties of sites sampled north of Inuvik in 2022. The site ID indicates the dominant vegetation type (see also second column) at each site and the replicate number. Site coordinates are given in decimal degrees, elevation in meters above sea level and active layer depth in centimetres below ground surface. Values for average soil temperature and associated standard deviation are given in degrees Celsius for two depth ranges: 0 – 20 cm and 30 – 50 cm, approximately representing topsoil & upper subsoil and lower subsoil, respectively. Water content is given in weight percent relative to soil wet weight for topsoil, upper subsoil and lower subsoil horizons.

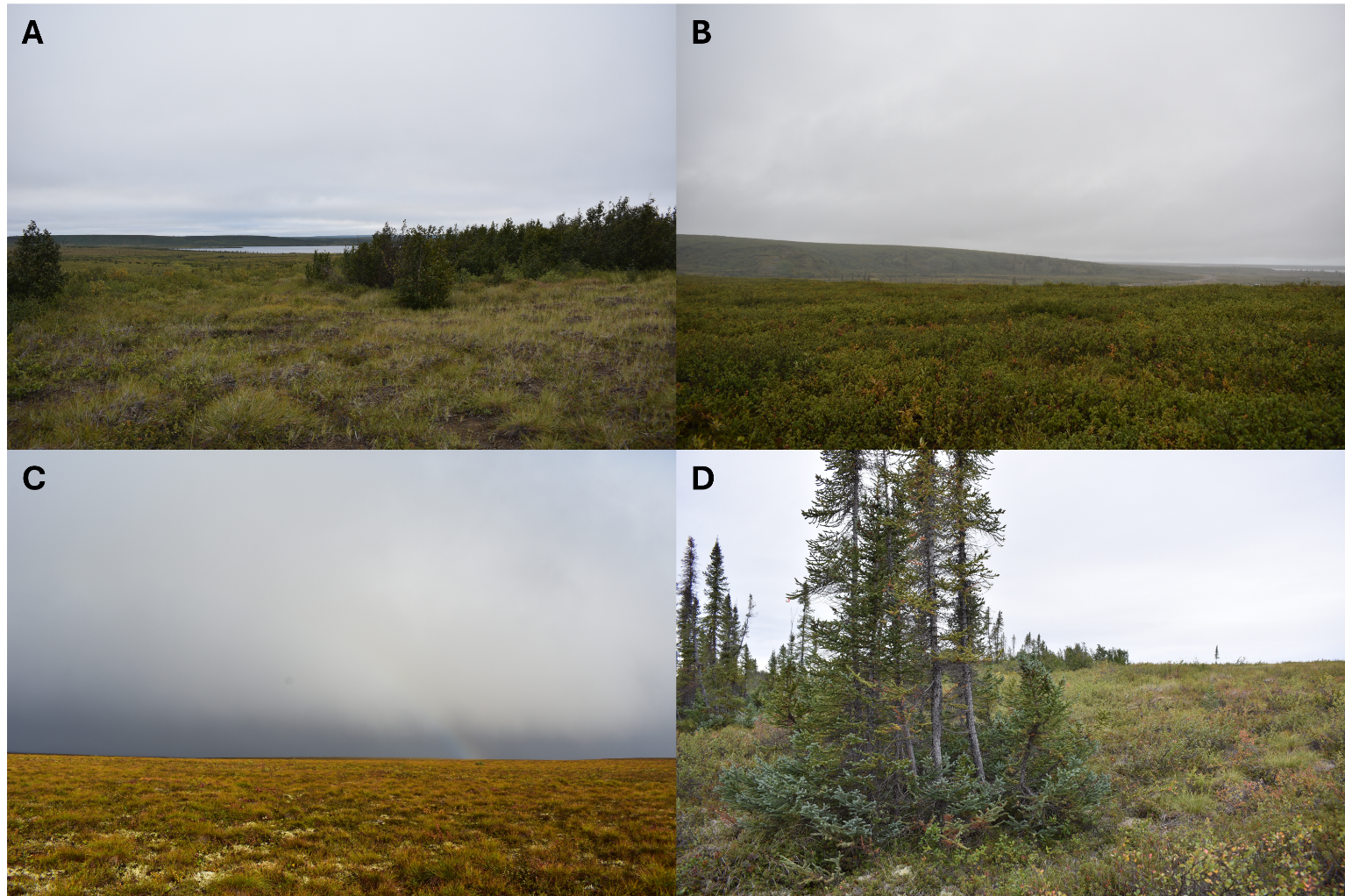


Figure S1: Photographs of research sites. Panels A – D depict the alder shrub, birch shrub, graminoid tussock and spruce tree sites, respectively.


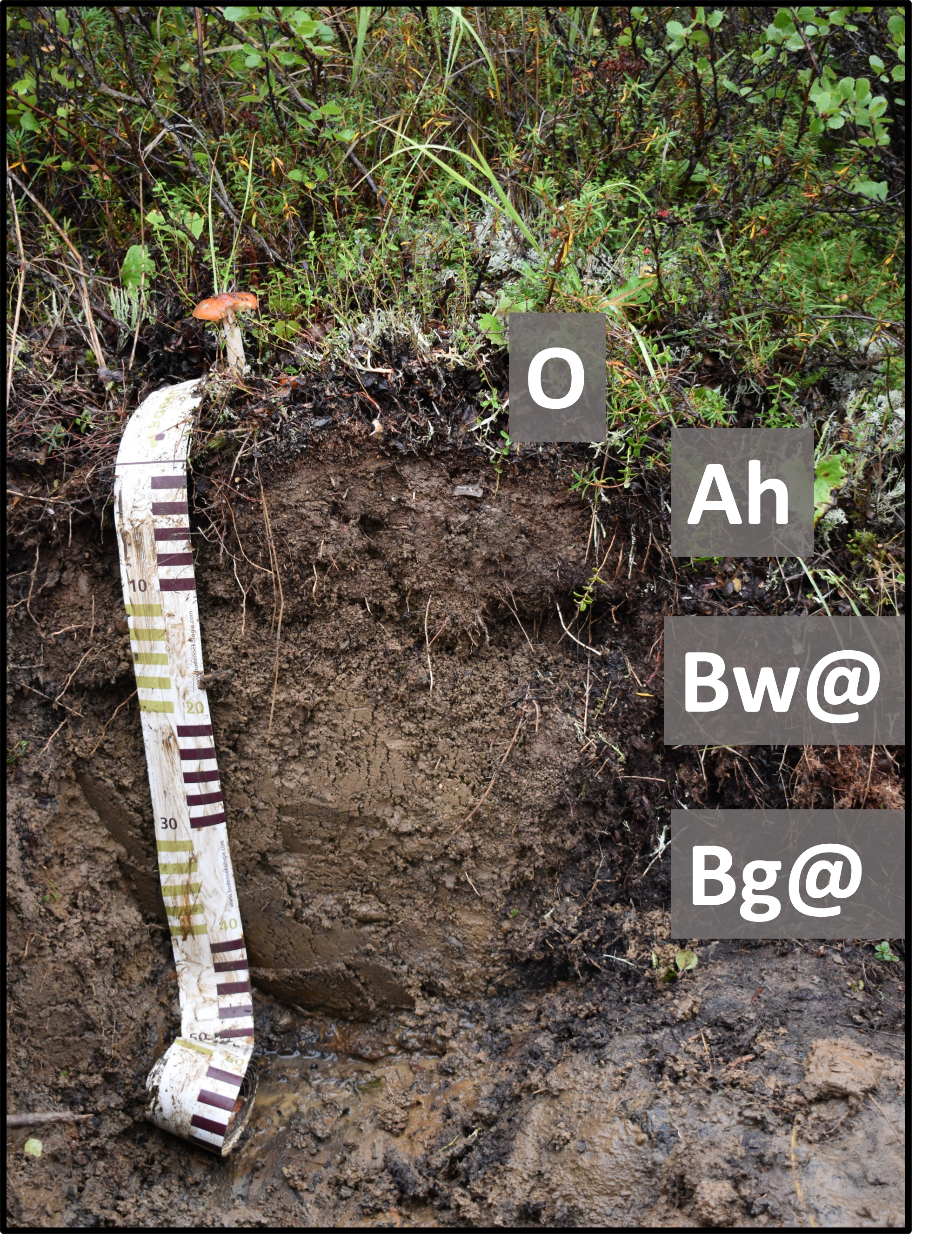


Figure S2: Photograph of an exemplary soil profile within our research area. The approximate locations of organic surface horizon (O), topsoil (Ah), upper subsoil (Bw@) and lower subsoil (Bg@) are indicated with white text.

**Table S2**: Bulk soil properties. General site properties are given as main site ID (based on dominant vegetation cover; see also Table S1), subsite ID (replicate number) and soil horizon. Following soil properties are given: pH, bulk density, total lignin content normalized to organic carbon and soil dry weight, lignin degradation proxy (Vd/Vl), total nitrogen (TN) and organic carbon content (OC), C/N, the amounts of free, occluded and mineral fraction in weight percent and soil texture including clay, silt and sand content in % and the soil texture class.

**Table S3**: Litter and organic horizon properties. General site properties are given as main site ID (based on dominant vegetation cover; see also Table S1), subsite ID (replicate number) and soil horizon. Following soil properties are given: total lignin content normalized to organic carbon and soil dry weight, lignin degradation proxy (Vd/Vl), total nitrogen content (TN), organic carbon content (OC) and C/N.

**Table S4**: Free fraction properties. General site properties are given as main site ID (based on dominant vegetation cover; see also table S1), subsite ID (replicate number), soil fraction and soil horizon. Following soil properties are given: pH, bulk density, total lignin content normalized to organic carbon and soil dry weight, lignin degradation proxy (Vd/Vl), total nitrogen (TN) and organic carbon content (OC), δ^13^C and C/N. Additionally, the amounts of lignin, nitrogen and carbon stored in the respective fraction are given as percentages normalized to the total amount in a given sample.

**Table S5**: Occluded fraction properties. General site properties are given as main site ID (based on dominant vegetation cover; see also table S1), subsite ID (replicate number), soil fraction and soil horizon. Following soil properties are given: pH, bulk density, total lignin content normalized to organic carbon and soil dry weight, lignin degradation proxy (Vd/Vl), total nitrogen (TN) and organic carbon content (OC), δ^13^C and C/N. Additionally, the amounts of lignin, nitrogen and carbon stored in the respective fraction are given as percentages normalized to the total amount in a given sample.

**Table S6**: Mineral fraction properties. General site properties are given as main site ID (based on dominant vegetation cover; see also table S1), subsite ID (replicate number), soil fraction and soil horizon. Following soil properties are given: pH, bulk density, total lignin content normalized to organic carbon and soil dry weight, lignin degradation proxy (Vd/Vl), total nitrogen (TN) and organic carbon content (OC), δ^13^C and C/N. Additionally, the amounts of lignin, nitrogen and carbon stored in the respective fraction are given as percentages normalized to the total amount in a given sample.
